# A ketogenic diet supplemented with medium-chain triglycerides enhances the anti-tumor and anti-angiogenic efficacy of chemotherapy on neuroblastoma xenografts in a CD1-nu mouse model

**DOI:** 10.18632/oncotarget.20041

**Published:** 2017-08-08

**Authors:** Sepideh Aminzadeh-Gohari, René Günther Feichtinger, Silvia Vidali, Felix Locker, Tricia Rutherford, Maura O’Donnel, Andrea Stöger-Kleiber, Johannes Adalbert Mayr, Wolfgang Sperl, Barbara Kofler

**Affiliations:** ^1^ Department of Pediatrics, Laura Bassi Centre of Expertise-THERAPEP, Research Program for Receptor Biochemistry and Tumor Metabolism, Paracelsus Medical University, Salzburg, Austria; ^2^ Clinical Nutrition Vitaflo International, Liverpool, United Kingdom; ^3^ Department of Pediatrics, Paracelsus Medical University, Salzburg, Austria

**Keywords:** neuroblastoma, ketogenic diet, medium-chain triglycerides, Warburg effect

## Abstract

Neuroblastoma (NB) is a pediatric malignancy characterized by a marked reduction in aerobic energy metabolism. Recent preclinical data indicate that targeting this metabolic phenotype by a ketogenic diet (KD), especially in combination with calorie restriction, slows tumor growth and enhances metronomic cyclophosphamide (CP) therapy of NB xenografts. Because calorie restriction would be contraindicated in most cancer patients, the aim of the present study was to optimize the KD such that the tumors are sensitized to CP without the need of calorie restriction. In a NB xenograft model, metronomic CP was combined with KDs of different triglyceride compositions and fed to CD1-nu mice *ad libitum*. Metronomic CP in combination with a KD containing 8-carbon medium-chain triglycerides exerted a robust anti-tumor effect, suppressing growth and causing a significant reduction of tumor blood-vessel density and intratumoral hemorrhage, accompanied by activation of AMP-activated protein kinase in NB cells. Furthermore, the KDs caused a significant reduction in the serum levels of essential amino acids, but increased those of serine, glutamine and glycine. Our data suggest that targeting energy metabolism by a modified KD may be considered as part of a multimodal treatment regimen to improve the efficacy of classic anti-NB therapy.

## INTRODUCTION

Neuroblastoma (NB) is the second most common extracranial solid malignancy in children, accounting for ∼15% of pediatric cancer mortality [1]. Favorable and unfavorable prognoses of NB are classified on the basis of the heterogeneity of genetic changes, histologic features, and patient age [2]. Owing to intense research efforts in NB therapy, including the application of multimodal therapeutic strategies, the survival rate has markedly improved in patients with low- and intermediate-stage NB [3]. However, despite these advances, the outcome for the high-risk NB group remains poor (< 50% long-term survival) [4]. Standard treatments for high-risk NB patients include several cycles of induction chemotherapy with subsequent surgical resection of the primary tumor. This is followed by high-dose consolidation therapy with autologous hematopoietic stem-cell transplantation and irradiation, and post-consolidation therapy to minimize residual disease [4]. Large numbers of adult survivors of childhood cancer have a high burden of morbidity, caused by side effects of standard cancer therapies that can persist or even develop months or years after treatment. The most commonly reported adverse health outcomes in adult survivors of pediatric malignancy include impaired pulmonary, auditory, cardiac, endocrine, psychosocial/cognitive and nervous system functions [5-8]. To address this challenge in NB therapy, addition of adjuvant treatment regimens may be opportune to improve the efficacy of anti-NB therapy, minimize its toxicity, and enhance patient quality of life.

In this regard, some key metabolic features of NB may be exploited to open a new less-toxic therapeutic frontier against this cancer. Various research studies have demonstrated that, similar to most solid cancers, NB shows a Warburg effect in energy production; that is, high glucose uptake and a preference to ferment glucose into lactate while only partially using mitochondrial oxidative phosphorylation (OXPHOS) [9-12]. Thus, in comparison to normal cells, a critical metabolic feature of NB is its low content of OXPHOS complexes [13].

Based on the high dependency on glucose and the OXPHOS deficiency in NB, we postulated that we could exploit the Warburg effect in NB cells by using a high-fat, low-carbohydrate or ‘ketogenic’ diet (KD) as part of a new multimodal NB therapy. Our assumption is that tumor cells exposed to KD would be forced to obtain their energy from fatty acid oxidation through OXPHOS. Because NB cells are less able to use fatty acids for ATP production than normal cells (because of their respiratory chain deficiency), they would have an energetic stress and therefore a survival disadvantage under a KD regimen.

A growing number of preclinical and clinical studies report that dietary intervention by KD is a potent anti-cancer therapy. It has been shown that KD not only improves the treatment efficacy of conventional therapies, but can also be safely applied in cancer patients [14-24]. Several animal studies, especially of brain tumors, demonstrate that KD has considerable anti-tumor effects [25-33], particularly in combination with calorie restriction [19, 34-36]. Part of the effect induced by KD is thought to be through its mimicking of a fasting phenotype via induction of ketosis. *In vivo* evidence shows that short-term starvation or fasting increases the efficacy of chemotherapy in various mouse cancer models [37, 38]. It has also been reported that fasting can ameliorate side effects caused by chemotherapy drug toxicity in patients [39, 40]. In agreement with these findings, our latest studies indicated that calorie restriction enhanced the effect of a long-chain triglycerides (LCT)-based KD on NB growth inhibition and survival in a xenograft model [41]. Additionally, the KD generated an even greater suppression of NB growth when it was combined with metronomic cyclophosphamide (CP) [42].

In particular, advanced-stage cancer patients can experience cancer-induced cachexia, which is an important reason for morbidity and mortality in cancer treatment [43-45]. In view of this phenomenon, calorie restriction therapy does not seem to be feasible for a number of cancer patients [21, 22]. The present study addresses this challenge by focusing on the optimal composition of KD to avoid calorie restriction as well as to enable lower doses of chemotherapy in NB treatment. Based on multiple benefits of medium-chain triglycerides (MCTs)-containing diets compared to pure long-chain triglycerides (LCTs)-based diets in terms of digestion and metabolism [46-50], we decided to compare the effects of an LCT-based KD versus a KD supplemented with 25% 8- and 10-carbon medium-chain triglycerides (LCT-MCT8 and LCT-MCT10) on metronomic CP therapy of NB xenografts. An MCT-supplemented KD was initially used as a therapeutic tool for refractory epilepsy in 1971 [51] and was first introduced into cancer studies in 1987, in which the diet was able to reverse the cachectic process and reduce the tumor weight in a colon adenocarcinoma model [52]. Only a few studies have since employed MCT-containing KDs for cancer therapy. Preclinical and clinical studies indicate that the diet is feasible in most patients and has anti-tumor effects in animal models [53-58]. In addition, it has been shown that MCTs have a higher anti-inflammatory effect compared to LCTs [50, 59, 60]. LCTs are incorporated first into chylomicrons, and then enter the lymphatic system to be oxidized in the liver. In contrast, MCTs are rapidly absorbed into the portal venous system [46, 47] and subsequently enter cells through phospholipid bilayers without the need of fatty acid transport proteins [48], and are then metabolized by mitochondria independent of carnitine [47].

Our studies reported here also evaluated the effects of the different KDs on blood glucose, ketone bodies and amino acids levels as well as parameters regarding tumor angiogenesis, hypoxia and metabolic stress.

## RESULTS

### Ketogenic dietary intervention with LCT-MCT8 effectively sensitizes NB xenografts to cyclophosphamide

**Figure 1:**
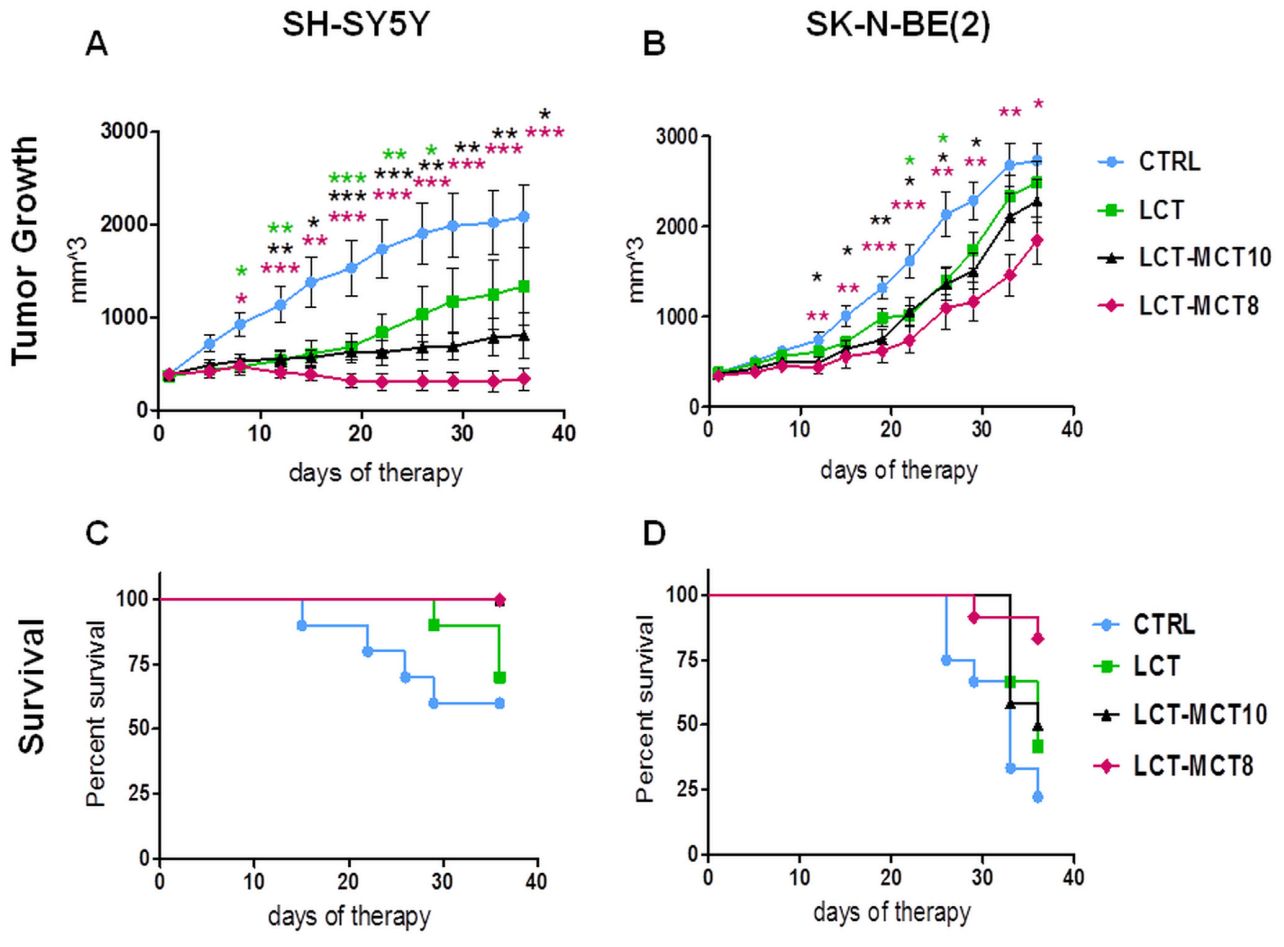
NB growth was most effectively inhibited by metronomic CP in combination with dietary intervention with LCT-MCT8 Growth suppression of **A.** SH-SY5Y and **B.** SK-N-BE(2) xenografts was most pronounced for metronomic CP combined with LCT-MCT8 KD compared to the other KDs and the CRTL diet. Kaplan-Meier survival curves for mice with **C.** SH-SY5Y and **D.** SK-N-BE(2) xenografts indicate prolonged survival of the LCT-MCT8 group. Values are given as mean ± SEM (CTRL, LCT and LCT-MCT10, *n* = 10-12; LCT-MCT8, *n* = 11-12). One-way ANOVA followed by Dunnett’s Multiple Comparison Test; * *p* ≤ 0.05; ** *p* ≤ 0.01; *** *p* ≤ 0.001; KDs in combination with metronomic CP vs CTRL in combination with metronomic CP. The statistical analysis for the survival curves was done with the Log-rank test (Mantel-Cox): SH-SY5Y xenograft, CTRL vs LCT, *p* = 0.4; CTRL vs LCT/MCT8, *p* = 0.02; CTRL vs LCT/MCT10, *p* = 0.02. SK-N-BE(2) xenograft, CTRL vs LCT, *p* = 0.09; CTRL vs LCT/MCT8, *p* = 0.002; CTRL vs LCT/MCT10, *p* = 0.07. When the tumor size reached the termination size some mice had to be sacrificed before the pre-determined end date of the therapeutic intervention. The tumor size data from the last measurement was kept in the calculation for the overall tumor growth rate at later time points.

To elucidate whether the sensitization of NB xenografts to low-dose chemotherapy by KD is influenced by the triglyceride composition, we treated NB-bearing mice with metronomic CP in combination with either an LCT-based KD or MCT-supplemented KDs (LCT-MCT8, LCT-MCT10). As a recent *in vitro* study reported that 10-carbon MCT, but not 8-carbon MCT, induced energy metabolism and mitochondrial activity in SH-SY5Y cells [61], we decided to use KDs either supplemented with 8-carbon or 10-carbon MCTs. Furthermore, we restricted the MCT content to 33% of the total fat content as very high amounts of MCTs in the diet can cause temporary gastrointestinal distress in humans. The daily dose we chose for CP was 40 mg/kg for mice with SH-SY5Y xenografts and 13 mg/kg for mice with SK-N-BE(2) xenografts, which led to a slight deceleration, but not inhibition, of tumor growth under the control diet (Figure 1, Supplementary Figure 1). Like we reported previously [42], the SK-N-BE(2) xenografts were more sensitive to metronomic CP than were the SH-SY5Y xenografts.

In addition to the delayed growth of the xenografts under metronomic CP, all KDs decelerated tumor growth further (Figure 1A, 1B). The dietary intervention with LCT-MCT8 was the most effective KD, producing significant inhibition of tumor growth in combination with metronomic CP, whereas LCT showed the least anti-NB effect (Figure 1A, 1B). In more detail, LCT-MCT8 induced near-complete growth inhibition of SH-SY5Y xenografts (Figure 1A), and led to tumor regression in 5 out of 11 cases (Supplementary Figure 1). LCT-MCT10 inhibited SH-SY5Y growth significantly over the whole intervention period (Figure 1A), but tumor regression was observed in only 1 of 10 cases (Supplementary Figure 1). LCT-MCT8 was the only KD that produced significant suppression of tumor growth in SK-N-BE(2) xenografts from day 12 till the end of the intervention (Figure 1B), albeit the effect was much less pronounced than the growth suppression on the SH-SY5Y xenografts. Compared to the control diet, intervention with the pure LCT-based KD significantly inhibited growth of the SH-SY5Y xenografts from day 8 to day 26 and reached a significant difference versus SK-N-BE(2) xenografts on day 22 and 26 only (Figure 1A, 1B).

Subsequently, 100% of mice with the SH-SY5Y xenografts survived over 36 days when treated with metronomic CP in combination with LCT-MCT8 or LCT-MCT10, while the survival rate for LCT and CTRL mice was 70% and 60%, respectively (Figure 1C). The survival rate for mice with SK-N-BE(2) xenografts was again highest in the LCT-MCT8 group compared to the other dietary groups. 84% of LCT-MCT8 mice survived the duration of therapy, whereas only 22%, 42% and 50% of mice in the CTRL, LCT and LCT-MCT10 groups, respectively, survived over the same period (Figure 1D).

### Effect of dietary intervention on glucose-ketone index

**Figure 2:**
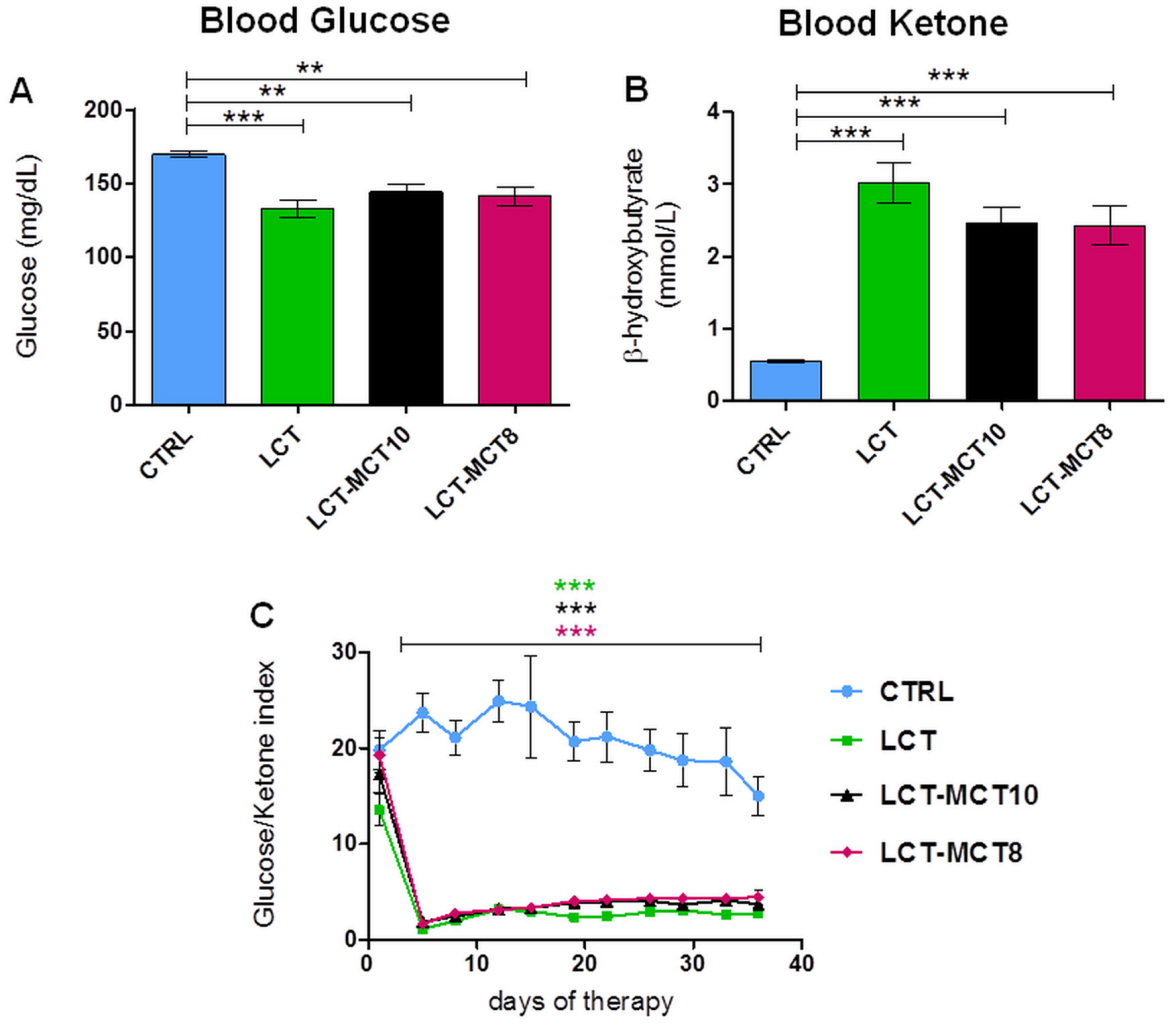
All KDs significantly lowered blood glucose levels and increased blood ketone body levels **A.** The mean blood glucose level significantly decreased and **B.** the mean BHB level significantly increased in KD-treated mice with NB xenografts over the duration of the experiment. **C.** The ratio of blood glucose to ketone levels was significantly lower in KDs over the duration of the experiment. Values are given as mean ± SEM (CTRL, LCT and LCT-MCT10, *n* = 22; LCT-MCT8, *n* = 23). One-way ANOVA followed by Dunnett’s Multiple Comparison Test; ** *p* ≤ 0.01; *** *p* <0.001; KDs in combination with metronomic CP vs CTRL in combination with metronomic CP.

To elucidate the effect of the dietary interventions on ketosis, we measured blood glucose and ketone body levels in the serum of all dietary groups. As expected, the blood glucose level in the KD groups, averaged over the whole therapy time, showed a significant reduction of about 20% compared to the CTRL group (Figure 2A; Supplementary Figure 3A, 3B). The serum ketone level, measured as the concentration of beta-hydroxybutyrate (BHB) and similarly averaged over the treatment duration, increased by at least 5.6-fold in mice fed the KDs versus the CTRL group (Figure 2B, Supplementary Figure 3C, 3D). The different KD compositions did not elicit significant differences in blood glucose and BHB levels.

The glucose-ketone index (GKI) is regarded as a better indicator of the efficacy of a metabolic therapy [62]. Over the entire period of the dietary intervention, the GKI was significantly reduced (p<0.001) in all KD groups compared to the CTRL group. Again, there was no significant difference in GKI among the different KD compositions.

We observed a decrease in the mean body weight of mice receiving the KDs during the first days of therapy, but this then stabilized and the weight was regained in most animals after 2-3 weeks (Supplementary Table 1; Supplementary Figure 4).

### Ketogenic dietary intervention alters amino acid levels and urea cycle metabolites

As the KDs contain only 50% of the protein compared to the CTRL diet (Supplementary Table 1), we tested if the KDs influence the amino acid (AA) levels in plasma and tumor tissues.

**Figure 3:**
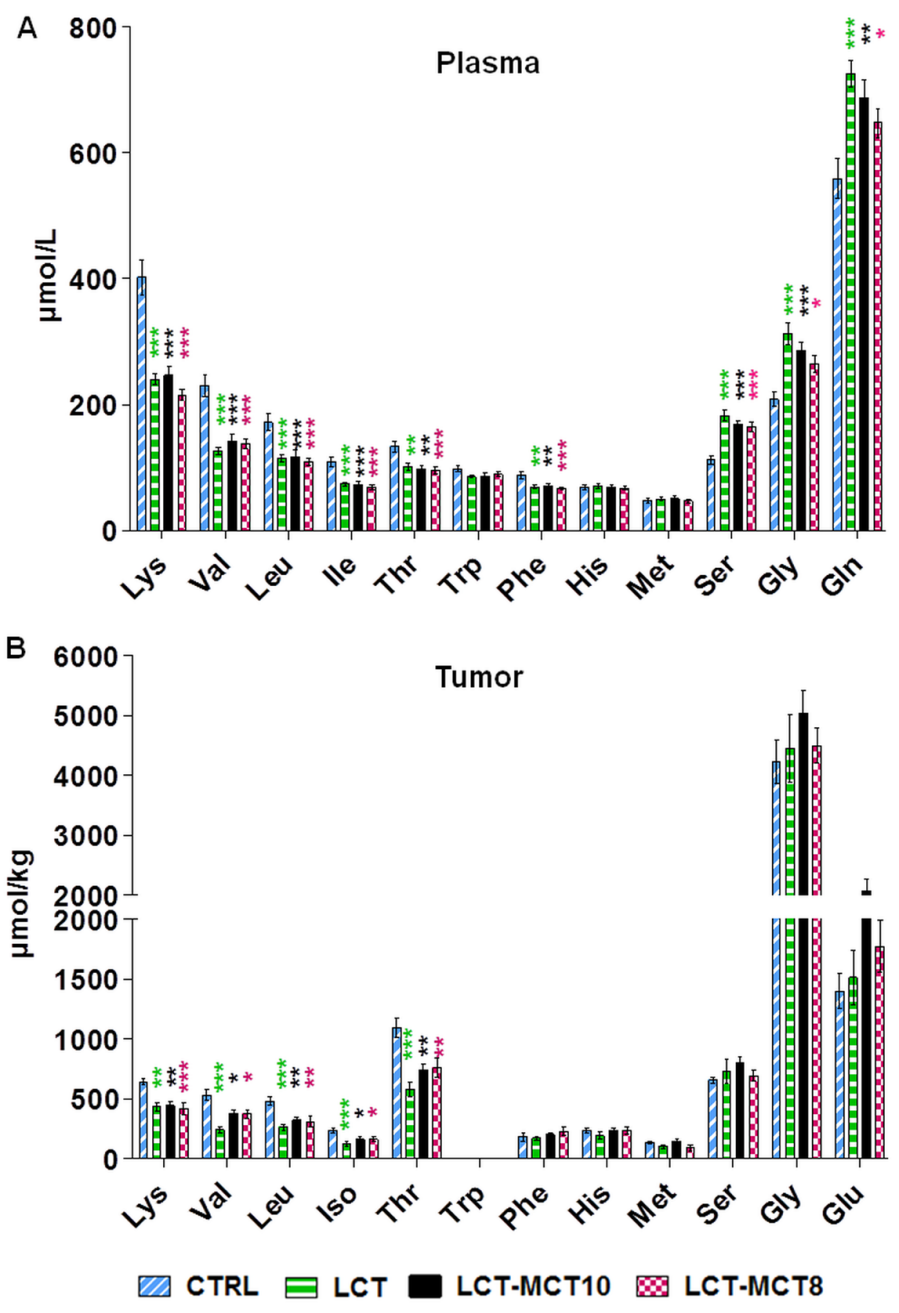
Amino acid levels in plasma and tumor tissue were affected by KDs Measurement of amino acid levels in **A.** plasma of mice with NB xenografts and in **B.** NB tumors (SK-N-BE(2)) revealed significantly lower levels of several essential amino acids and higher levels of the non-essential amino acids serine, glycine and glutamine in the KD groups. Values are given as mean ± SEM (plasma, *n* = 16; tumor, *n* = 8). One-way ANOVA followed by Dunnett’s Multiple Comparison Test; * *p* ≤ 0.05; ** *p* ≤ 0.01; *** *p* ≤ 0.001; KDs in combination with metronomic CP vs CTRL in combination with metronomic CP. Tryptophan was below the detection limit in tumor tissues.

Plasma AA measurement showed significantly lower levels of the essential AAs lysine, valine, leucine, isoleucine, threonine and phenylalanine in the KD groups compared to the CTRL group (Figure 3A). Similar changes of essential AAs were detected in the NB xenografts (Figure 3B), with the exception that phenylalanine levels were found to be similar in all dietary groups, and tryptophan was not detectable in tumors. Among the non-essential amino acids, significantly higher levels of glutamine, serine, and glycine were found in the plasma of the KD groups compared to the CTRL group (Figure 3A; Supplementary Figure 5). However, in the tumors, no differences in non-essential AAs were observed among the various dietary groups (Figure 3B; Supplementary Table 2 and 3).

**Figure 4:**
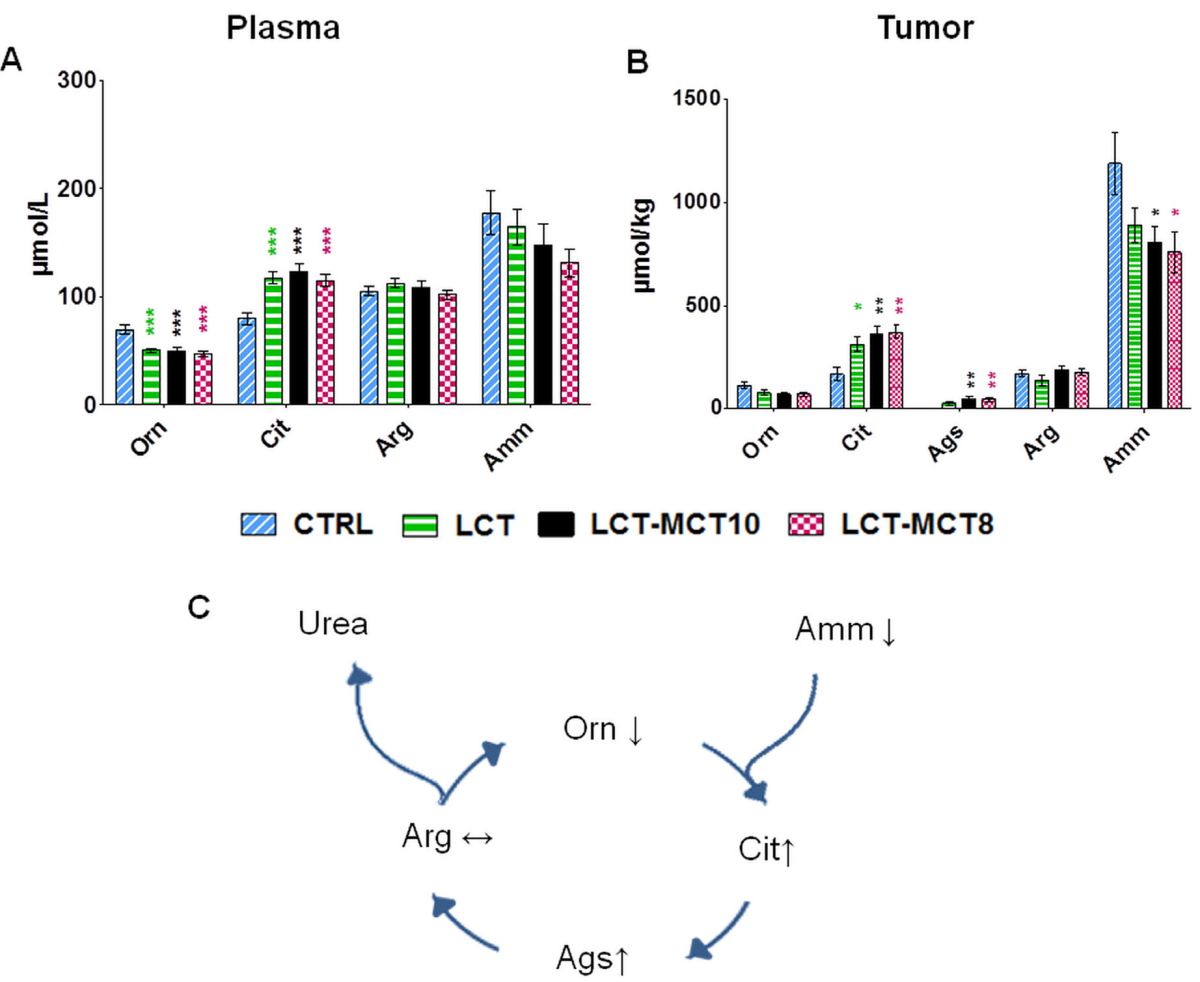
Levels of urea cycle metabolites were altered by KDs The level of ornithine (Orn) was significantly reduced in the **A.** plasma of mice with NB xenografts, and the level of citrulline (Cit) was significantly increased in both the **A**. plasma of mice with NB xenografts and in **B.** tumor tissue (SK-N-BE(2)), in the KD groups. Argininosuccinate (Ags) was only detectable in tumors of KD-treated mice. Ammonia (Amm) levels were lower in the KD groups compared to the CTRL group. **C.** Illustration summarizing the changes in urea metabolites induced by KDs. Values are given as mean ± SEM (plasma, *n* = 16; tumor, *n* = 8). One-way ANOVA followed by Dunnett’s Multiple Comparison Test; * *p* ≤ 0.05; ** *p* ≤ 0.01; *** *p* ≤ 0.001; KDs in combination with metronomic CP vs CTRL in combination with metronomic CP.

Urea cycle metabolites were also altered by the KDs (Figure 4). Plasma ammonia levels tended to be lower in the KD groups compared to the CTRL group. The concentration of ammonia was also lower in the tumors of KD-fed mice, reaching significance in the MCT-supplemented groups (Figure 4). Among the four metabolites of the urea cycle, plasma ornithine levels were significantly lower in the KD groups compared to the CTRL group, while citrulline was significantly higher both in plasma and xenografts. Remarkably, in tumor tissues, argininosuccinate, the intermediate between citrulline and arginine in the cycle, was detectable in the KD groups only. Interestingly, arginine levels did not differ in plasma or tumor tissue among the different dietary groups (Figure 4; Supplementary Table 2 and 3).

### Low dietary protein does not affect NB growth

The observed changes in plasma and tumor AA levels in the KD groups compared to the CTRL group generated two questions. First, were the altered AA levels in the KD groups caused by cross-talk between fat- and protein-metabolic pathways or were they simply a consequence of the different protein contents in the KD (8%) and CTRL diet (16%). Second, could a lower protein content in the CTRL diet influence NB growth. To answer these questions, mice with xenografts were treated with metronomic CP and fed two different control diets, one containing 16% (CTRL) and the other 8% (CTRL-8%) protein (Supplementary Table 1). Interestingly, the growth of NB xenografts in mice given either the CTRL or CTRL-8% diet showed no difference, whereas the LCT-MCT8 diet caused inhibition of NB growth like that shown in Figure 1 (Supplementary Figure 2A, 2B). Compellingly, the plasma levels of AAs and urea cycle metabolites of mice exposed to the lower protein content of the CTRL-8% diet did not differ from those of mice fed the CTRL diet (Supplementary Figure 2C, 2D).

### Ketogenic dietary intervention promotes the anti-angiogenic effect of cyclophosphamide

**Figure 5:**
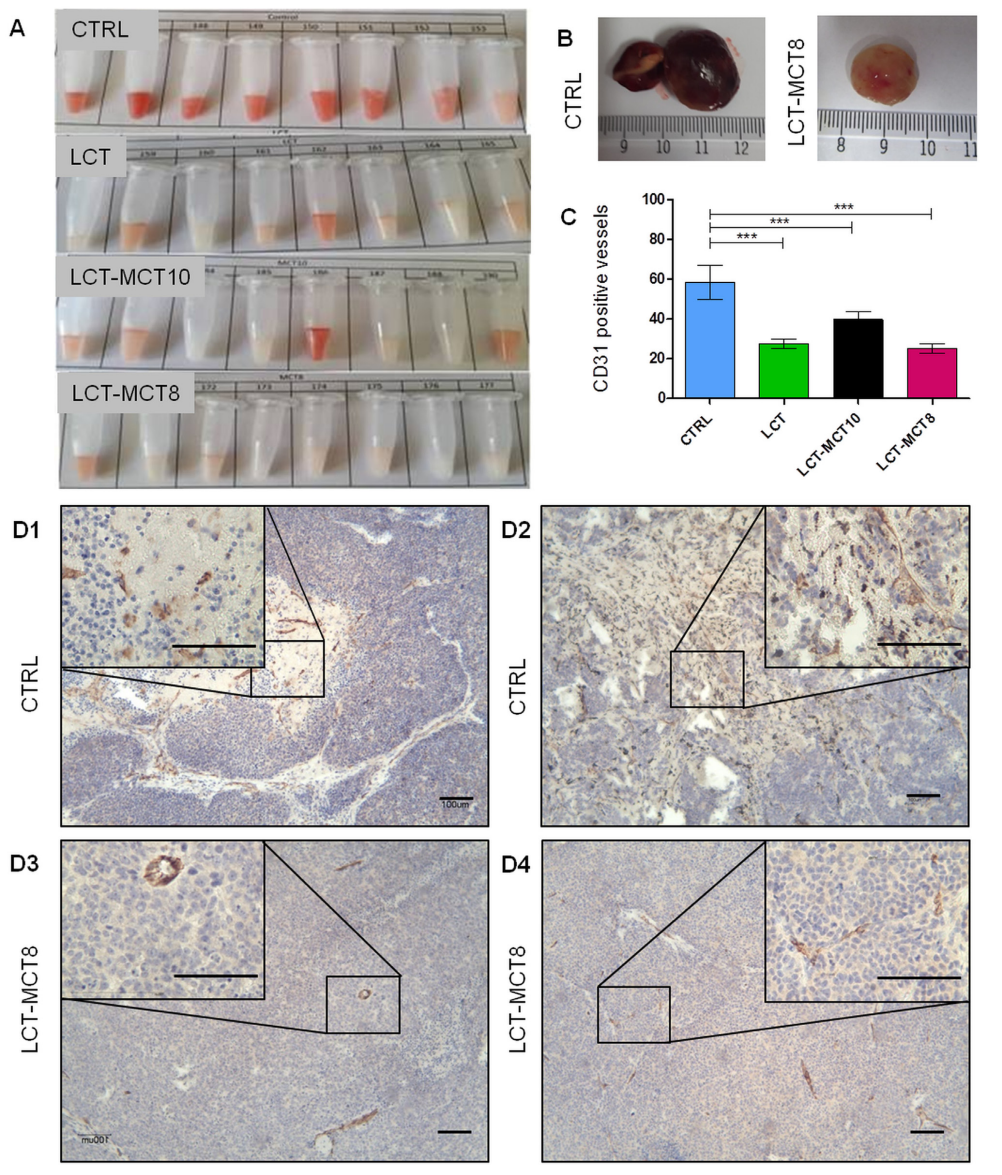
Macroscopic evaluation of intratumoral hemorrhage and microscopic evaluation of tumor vascularization revealed an anti-angiogenic effect of KDs **A.** SK-N-BE(2) xenograft tumor homogenates and **B.** whole tumors showed lower levels of intratumoral hemorrhage in the KD-treated groups compared to the CTRL group. **C.** Quantification of CD31-positive vessels (counted in five random high-power fields, 100-fold magnification) revealed a significant reduction in vessel density. Representative immunohistochemical staining for the endothelial marker CD-31 in four tumors from the **D1, D2** CTRL and **D3, D4** LCT-MCT8 groups revealed differences in blood vessel morphology and density. Scale bar = 100 µm. Values are given as mean ± SEM (*n* = 7-12). One-way ANOVA followed by Dunnett’s Multiple Comparison Test; *** *p* ≤ 0.001.

An angiogenic phenotype is a key risk factor in poor prognosis in NB, and one we observed especially in the SK-N-BE(2) xenografts. All KDs synergistically enhanced the anti-angiogenic efficacy of metronomic CP, with no clear superiority of any one KD. The combination of a KD with metronomic CP strikingly reduced intratumoral hemorrhage in SK-N-BE(2) tumors (Figure 5A, 5B). Quantification of CD31 staining (Figure 5C) revealed a significant reduction of vessel density in the xenografts derived from KD-fed mice compared to CTRL mice. The reduction of angiogenesis was also accompanied by enhanced blood vessel maturation (Figure 5D).

### Carbonic anhydrase IX levels are associated with necrosis

**Figure 6:**
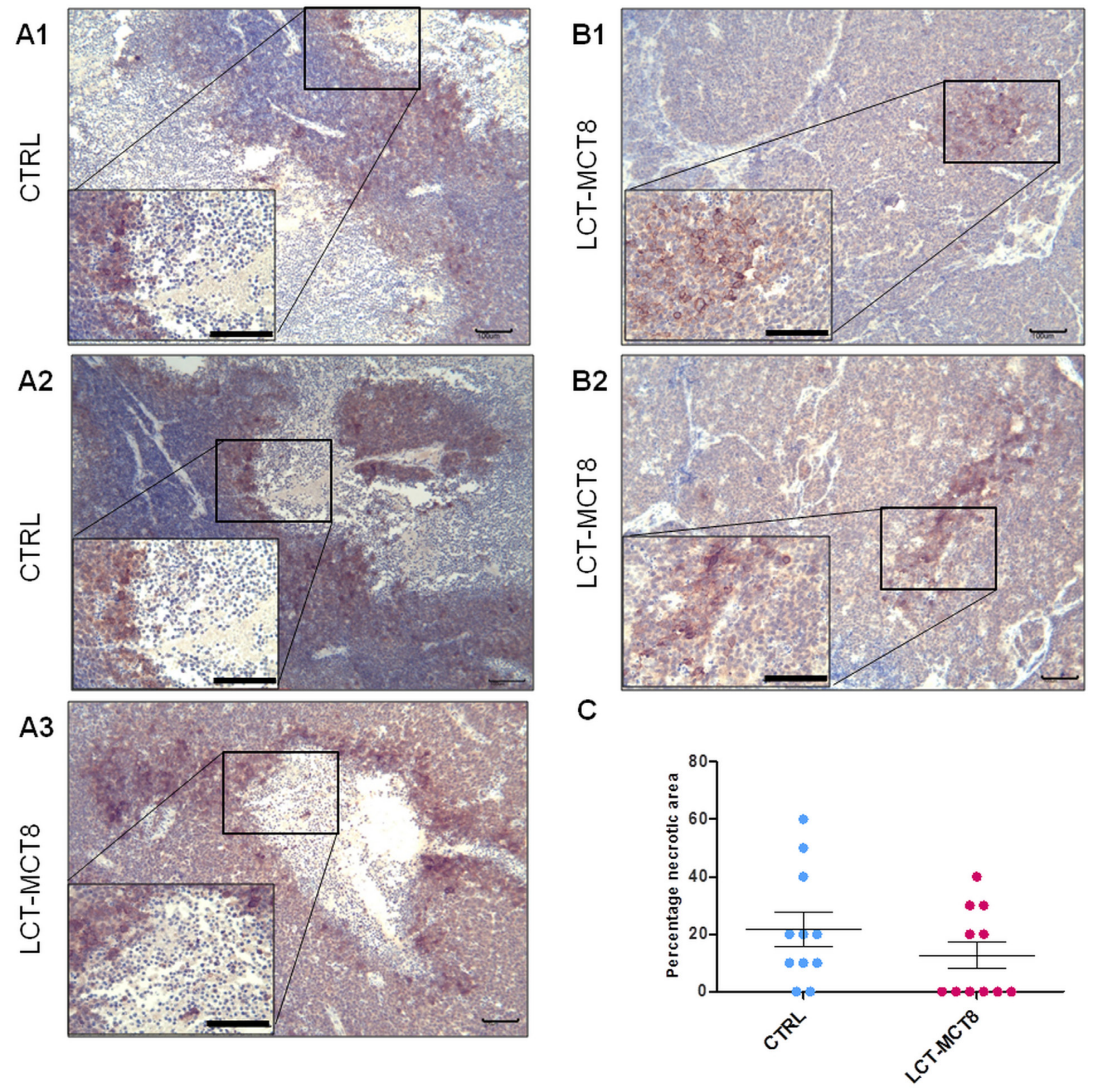
Expression of carbonic anhydrase IX (CAIX) was associated with necrosis **A1.**-**A3.** Immunohistochemical staining for CAIX shows the expression of CAIX at the border of necrotic areas in CTRL and LCT-MCT8 groups. **B1.**, **B2.** Expression of CAIX in non-necrotic tumors in the LCT-MCT8 KD group was lower and focal. **C.** The mean of the percentage of necrotic areas in SK-N-BE(2) tumors was higher in the CTRL group compared to the LCT-MCT8 group, although this difference was not significant (*n* = 11). Scale bar = 100 µm.

Because anti-angiogenic treatment may lead to an increase in hypoxia [63, 64], we analyzed the level of carbonic anhydrase IX (CAIX), a commonly used marker of hypoxia [65, 66]. Immunohistochemical analysis of CAIX indicated no significant difference between SK-N-BE(2) tumors with high vessel density in the CTRL group compared to tumors with less vascularization in the LCT-MCT8 group. Consistently, expression of CAIX was observed around necrotic areas in both the LCT-MCT8 and CTRL groups (Figure 6A1-6A3).

The number of necrotic tumors was higher in the CTRL (81%) than in the LCT-MCT8-fed group (45%) (Figure 6C). Focal expression of CAIX was observed mainly in the smaller and non-necrotic tumors of the LCT-MCT8 group (Figure 6B1, 6B2).

### Ketogenic dietary intervention gives rise to energy stress in NB

**Figure 7:**
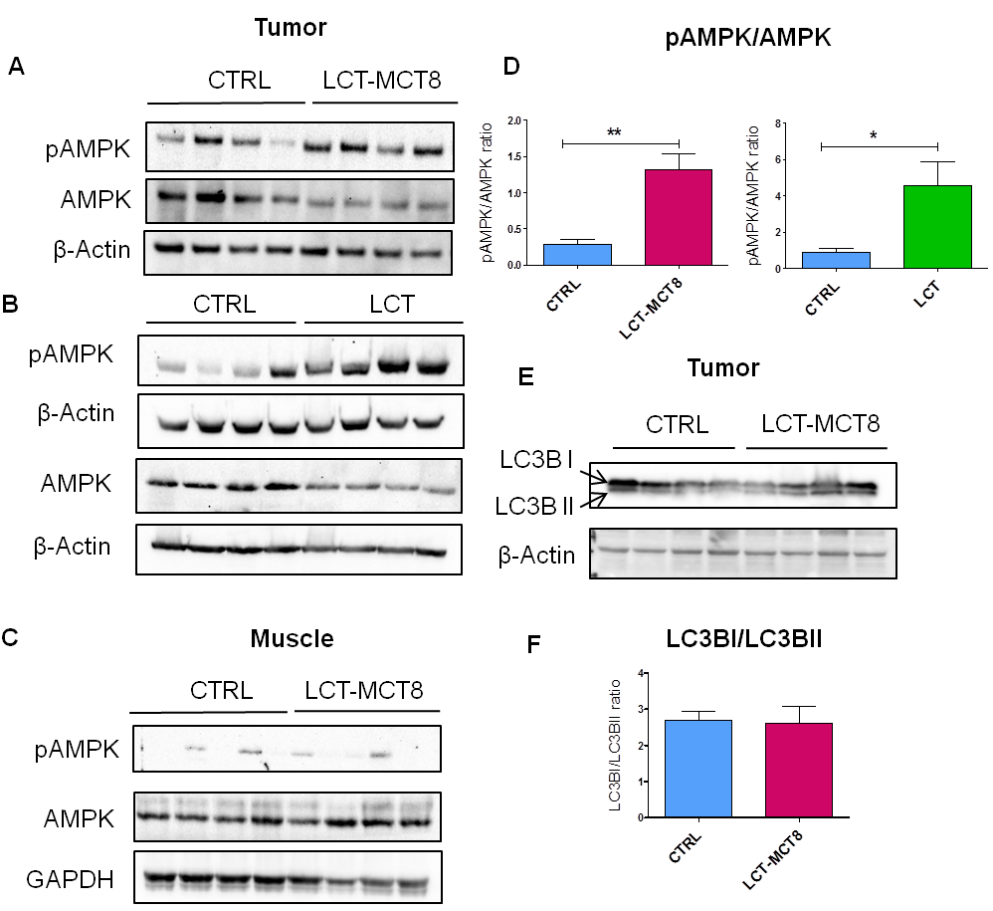
Western blot analysis of SK-N-BE(2) xenografts showed energy stress and lack of autophagy in LCT-MCT8 group compared to CTRL Western blot analysis for pAMPK, AMPK and β-Actin in SK-N-BE(2) tumors in the **A.** LCT-MCT8 and **B.** LCT groups versus the CTRL group showed activation of AMPK in the KD groups. **C.** Western blot analysis for pAMPK, AMPK and GAPDH in muscle tissues of mice revealed no difference between the LCT-MCT8 and CTRL groups. **D.** The ratio of pAMPK to AMPK band intensities indicates higher levels of the activated form of AMPK in the KD groups. The pAMPK and AMPK bands were normalized to the β-Actin levels. **E.** Western blot analysis for LC3B and β-Actin in SK-N-BE(2) tumors as well as **F.** the ratio of LC3BI to LCTBII band intensities revealed no difference in autophagy levels between the LCT-MCT8 and CTRL groups. Values are given as mean ± SEM. Unpaired *t* test; * *p* ≤ 0.05; ** *p* ≤ 0.01.

Due to OXPHOS deficiency and a low capability of ketone body utilization in NB [41], it is expected that KDs will induce a bioenergetic challenge for tumors, but not for normal tissue. To address this hypothesis, we evaluated the activation of the cellular energy sensor AMP-activated protein kinase (AMPK) in SK-N-BE(2) tumors from mice fed different diets. Western blot analysis revealed elevated levels of the activated (i.e., phosphorylated) form of AMPK in SK-N-BE(2) tumors in the LCT and LCT-MCT8 groups compared to the CTRL group (Figure 7A, 7B, 7D). In contrast, the pAMPK to AMPK ratio in mouse muscle was not influenced by the KDs (Figure 7C).

Tumor cells can trigger autophagy in response to cellular stress, including increased metabolic and energy demands [67]. Based on the observed energy stress and the significant reduction of essential AAs in the tumors of KD-group mice, we explored whether SK-N-BE(2) tumors exposed to KDs underwent autophagy, for example to maintain precursors for cellular biosynthesis. Western blot analysis of Light chain 3 (LC3B) indicated no remarkable change between the ratio of LC3B-I and LC3B-II in the xenografts of the LCT-MCT8 group versus the CTRL group (Figure 7E, 7F), suggesting there was no up-regulation of autophagy in the KD-exposed tumors.

## DISCUSSION

Our previous preclinical studies on NB support the efficacy of KD as a non-toxic, complementary cancer therapy that sensitizes tumor cells to chemotherapy, particularly in combination with calorie restriction [42]. Calorie restricted KD has also been used as a complementary therapy for treatment of few brain cancer patients [16, 24], however, in clinical practice, calorie-restricted KD may not be offered to the majority of cancer patients [21, 22], especially those with cachexia [43, 44].

Only a few studies have given more than nominal attention to the triglyceride composition of KDs. In the present study, we used KDs supplemented with MCTs of different lengths to try to enhance the potency of low-dose chemotherapy in NB compared to our previous KD formulation [42]. In addition, to achieve the best possible effects of the KDs, we increased the fat content of the diets from 57% in our previous study [42] to 74.6% in the present study. Our data revealed that *ad libitum* KD supplemented with 25% MCT8 was as effective as the previously used LCT-only KD in combination with calorie restriction [42] in sensitizing NB to low-dose chemotherapy. In agreement with our new findings, KD has been shown to sensitize malignant glioma to radiation therapy and lung cancer to radio-chemo-therapy [27, 68]. Furthermore, dietary intervention with MCTs (80% of the energy) in preclinical studies on murine colon adenocarcinoma reduced both tumor proliferation and cancer cachexia [52, 56, 57, 69]. An omega-3/MCT-supplemented KD also delayed the growth of gastric cancer xenografts [54]. However, the growth of human colon xenografts was equally delayed by omega-3/MCT- and LCT-based KDs [53]. In addition, a high-fat, low-carbohydrate, moderate protein diet supplemented with MCT (60% fat (30% MCT)) was able to mimic the metabolic and anti-tumor effects of the KD and resulted in a reduction of glioblastoma progression and an increase of survival in an orthotopic xenograft model to a similar extend as a classical KD (fat:carbohydrate plus protein ratio of 8:1) [58]. The MCT supplemented diet had a lower total fat content than the KD. In our study we used a similar ketogenic ratio in the LCT and MCT supplemented diets and therefore the MCT supplementation led to a more pronounced anti-tumor effect on the neuroblastoma xenografts compared to the LCT-only diet. The mechanism of the higher efficacy of LCT-MCT8 compared to other KDs on NB growth in the present study remains elusive and will need further exploration. A recent *in vitro* study reported that 10-carbon MCT, but not 8-carbon MCT, induced energy metabolism and mitochondrial activity in SH-SY5Y cells [61], which might provide a partial explanation for the differing anti-NB properties between LCT-MCT10 and LCT-MCT8 in our study.

The xenografts of the TP53 wild-type, non-*MYCN*-amplified SH-SY5Y cells were considerably more sensitive to KD than were the SK-N-BE(2) xenografts, which carry *MYCN* amplification, TP53 mutation (p.C135F) and chromosome 1p loss of heterozygosity [70]. A differential response of cancer cells with different genetic alterations has also recently been reported for melanoma. In contrast to the inhibition of proliferation of NB xenografts observed in the present study, BRAF V600E-mutated melanoma cells showed increased proliferation upon dietary intervention with an LCT-based KD (75% fat), whereas NRAS Q61K-mutated and wild-type melanoma cells were not affected [71]. However, in a feasibility trial with a limited number of patients with advanced malignancies, a BRAF-positive melanoma patient seemed to benefit most from consumption of ketogenic diet after BRAF-inhibitor resistance [72]. In addition, Liśkiewicz et al. also observed that long-term KD (79% fat content) increased renal tumor growth in a rat model of Tuberous Sclerosis [73]. Furthermore, we recently reported that KD is not feasible as a therapy in a CD-1 nu/nu mouse model of renal cell carcinoma with features of Stauffer’s syndrome [74]. Therefore, it is of utmost importance to evaluate the effect of KDs in preclinical studies on specific types of tumors and also to take into account the different genetic alterations before considering clinical studies, as differences in the response of tumor growth to dietary intervention between mice and humans cannot be excluded.

We observed changes in the levels of specific AAs in both the plasma and the tumor in KD-fed mice but not in mice fed a control diet matching the low protein content of the KDs. These findings suggest that the high fat content, not the low protein content, of the KDs is causing specific alterations to the AA profile. The most consistent and pronounced decreases of serum AAs induced by the KDs were observed for essential AAs and urea cycle metabolites, whereas the plasma levels of serine, glycine and glutamine were elevated.

In agreement with our observation on AA profiles, Roberts et al. detected similar changes of AA plasma levels in rats given a KD (70% fat, 20% protein), as did Douris et al. in mice fed a KD (79% fat, 9% protein) [75, 76]. In contrast to our results for mice bearing NB xenografts, reduction of dietary protein (to 7%) in mice bearing prostate and breast cancer xenografts caused inhibition of tumor growth [77, 78].

The impact of the KD on down-regulation of AA catabolism [76] might contribute to its previously reported anti-cachexia effects [52, 56, 57, 69, 79]. The molecular mechanism explaining the effects of the KD on cancer growth is just beginning to be identified and the altered AA profile should be considered as a potential underlying anti-tumor mechanism of KD.

The fact that the KDs were less effective on the *MYCN*-amplified SK-N-BE-(2) xenografts compared to the non-*MYCN*-amplified SH-SY5Y tumors might inter alia be related to the fact that the MYC transcription factor enhances glutamine uptake and is a driver of glutamine-dependent tumors [80].

The higher level of AMPK activation measured in tumors of the KD groups, but not in normal tissue, provides evidence that NB cells experience metabolic stress during the dietary intervention, such that KD selectively sensitizes the NB cells to classic low-dose chemotherapy. The reduced supply of nutrients and subsequent negative energy balance resulting from the KD-induced lower blood vessel density might be another trigger for AMPK activation in NB cells.

A high level of angiogenesis and poor blood vessel formation are hallmarks of aggressive NB [81, 82]. Therefore, anti-neovascularization strategies are considered one option for combating NB [83-86]. Our previous study indicated that metronomic CP has an anti-vascularization effect on NB [42]. Interestingly, in the present study, we observed that the MCT-supplemented KDs strongly fortified the anti-angiogenic effect of cyclophosphamide. In agreement with our findings, an anti-angiogenic effect of calorie-restricted or *ad libitum* KD has been reported in brain and gastric cancer models [35, 54, 87, 88].

Anti-angiogenic therapy can lead to an imbalance between the high oxygen demands of rapidly proliferating cancer cells and the oxygen supply. Therefore, during treatment with an anti-angiogenic approach, tumors may experience hypoxia. CAIX is expressed in specific types of tumors as a mediator of the complex response to the low-oxygen environment [66]. In NB, a correlation between up-regulation of CAIX expression and poor survival, particularly in *MYCN*-amplified tumors, has been shown [89, 90]. Our study revealed that the strong inhibition of angiogenesis by the KD combined with low-dose cyclophosphamide did not stimulate a hypoxic response in NB. In accordance with this observation it has been shown that KD even reduce expression of CAIX and hypoxia inducible factor 1-alpha which is accompanied by reduction of tumor microvasculature in a mouse glioma model [88]. In contrast, reduction of angiogenesis by anti-vascular endothelial growth factor (VEGF) treatment induced hypoxia, which in turn promoted the growth and invasiveness of glioblastoma and colon cancer [91, 92].

Based on our findings in the present study, we hypothesize that enrichment of an *ad libitum* KD with specific triglycerides can increase the efficacy of NB therapy similar to that achieved with calorie-restricted KDs. Furthermore, such lipid-modified KDs may enable to use reduced doses of chemotherapy, according to the synergy we observed here between the KDs and the metronomic CP treatment.

## MATERIALS AND METHODS

### Cell lines

Two NB cell lines, namely SH-SY5Y (ATCC CRL-2266) and SK-N-BE(2) (ATCC CRL-2271), were selected for the xenograft studies. SH-SY5Y is a TP53 wild type, non-*MYCN*-amplified cell line with no chromosome 1p loss of heterozygosity. The SK-N-BE(2) cell line is amplified for *MYCN*, carries a TP53 mutation (p.C135F), and is heterozygous for loss of chromosome 1p [70]. Expression of MYCN protein in these cell lines was reported earlier [42]. NB cells were cultured under standard conditions, as described previously [41].

### Animal models and sample preparation

*In vivo* experiments were performed in accordance with the Salzburg Animal Care and Use Committee (Study approval no. 20901-TVG/115/6-2016). Animals were maintained under pathogen-free conditions. Xenografts were established on the right flanks of 5- to 6-week-old female CD-1 nude mice (Charles River, Wilmington, MA, USA). A 200-μl mixture of NB cells (2 ×10^7^ cells per mouse) in serum-free medium suspended (1:1) in matrigel (BD Bioscience, Franklin Lakes, NJ, USA) was subcutaneously injected. When the tumor size reached approximately 300 mm^3^, oral metronomic CP treatment was started and the mice were randomized into therapy groups (CTRL, CTRL-8%, LCT, LCT-MCT10 and LCT-MCT8; n = 10-12 per group). The animals were group-housed and had unlimited access to the food. Food intake per mouse was calculated from the average of the whole cage. Tumor volume, body weight, blood glucose and ketone body levels (beta-hydroxybutyrate) were monitored twice weekly [41]. At 36 days or when termination criteria were met (health status, tumor ulceration or volume of 2500 mm^3^), the mice were anesthetized with intraperitoneal injection of a solution (10 µl per gram body weight) composed of ketamine 20.5 mg/ml, xylazine 5.4 mg/ml and acepromazine 270 µg/ml in saline. Blood from the mice was taken by cardiac puncture and collected in BD microtainer tubes (Product Number: 365986BD Biosciences, Austria). The tubes were centrifuged at 10,000×g for 90 seconds at room temperature, and plasma was collected and snap frozen. Tissue samples were snap frozen in liquid nitrogen for further molecular analysis. For histological analysis, tumor slices were formalin-fixed and paraffin-embedded.

### Food composition, energy content and metronomic cyclophosphamide (CP)

Mice were fed different diets *ad libitum* (Supplementary Table 1). All diets were fortified with the same level of vitamins and mineral supplements. Cyclophosphamide (Sigma, St Louis, MO, USA) was given orally through the drinking water according to the study of Man et al. [93]. For SH-SY5Y-bearing mice, an oral dose of 40 mg/kg/day was administered [42]. Because of the higher sensitivity of SK-N-BE(2) xenografts to CP [42], an oral dose of 13 mg/kg/day was given to SK-N-BE(2)-bearing mice.

### Amino acid profiling

AA profiles were obtained from plasma and tumor tissue of mice with NB xenografts. Snap-frozen tumor tissues were homogenized in 5-fold (w/v) PBS using an Ultra-Turrax homogenizer (IKA, Staufen, Germany) and then homogenized with a motor-driven Teflon-glass homogenizer (Potter S, Braun, Melsungen, Germany). The homogenates were centrifuged at 16,000×g for 15 min at 4 °C and supernatants were collected. Then, AA levels of 100 µl plasma and tumor tissue supernatant were measured by a Biochrom 30 + Amino Acid Analyzer Physiological System (Biochrom Ltd.), as described previously [94].

### Immuno-histochemical staining and analysis

Immunhistological staining was performed using 4-μm deparaffinized tumor sections of SK-N-BE(2) xenografts. Staining with anti-msCD31 (Abcam, Cambridge, UK) and anti-CAIX (BioScience Slovakia) antibodies was carried out at a dilution of 1:100, as described previously [95]. For detection, an EnVision kit (Dako, Vienna, Austria) was used according to the manufacturer’s instructions. Blood-vessel densities were scored by counting the number of positively stained vessels on 5 random high-power fields (100-fold magnification) from 7-12 tumor samples of each dietary group. The level of tumor necrosis was evaluated in anti-CAIX-stained as well as hematoxylin-eosin stained samples.

### Western blot analysis

SK-N-BE(2) tumor and mouse muscle tissues (50-100 mg) were homogenized with an Ultra-Turrax homogenizer (IKA, Staufen, Germany) in 10-fold extraction buffer (20 mM Tris-HCl, pH 7.6, 250 mM sucrose, 40 mM KCl, 2 mM EGTA) and then homogenized with a motor-driven Teflon-glass homogenizer (Potter S, Braun, Melsungen, Germany). The homogenates were centrifuged at 600×g for 10 min at 4°C. A total of 20 µg protein was used for Western blot analysis, as previously described [95]. The following primary antibodies were diluted in TBS-T containing 10% western blocking reagent (Roche): 1:1000 anti-LC3 (MBL International Corporation, Vienna, Austria), 1:1000 Phospho-AMPKα (Thr172) (Cell Signaling Technologies (#2535), Frankfurt, Germany), 1:1000 anti-AMPK (Cell Signaling Technologies (#2532), Frankfurt, Germany), 1:5000 anti-beta Actin (Abcam, Cambridge, UK) and 1:5000 anti-GAPDH (Trevigen, Vienna, Austria). Horseradish peroxidase-labeled secondary antibodies were used (Dako, Vienna, Austria). Detection and quantification of band intensities was performed using Image Lab Software 5.2.1 (Bio-Rad) and normalized to the corresponding loading control.

### Statistics

Statistical analysis was performed using Prism 5 (GraphPad-M3 Software, USA). Data are given as mean ± SEM. Significance was determined by Student's t test (unpaired test) or one-way ANOVA (multiple comparisons two-tailed Dunnett's test). The statistical analysis of the survival curves was done with the Log-rank test (Mantel-Cox).

## SUPPLEMENTARY MATERIALS FIGURES AND TABLES



## Abbreviations

AA: amino acid
AMPK: AMP-activated protein kinase
CP: cyclophosphamide
CAIX: carbonic anhydrase IX
CTRL: standard control diet
KD: ketogenic diet
LC3B: Light chain 3
LCT: long-chain triglyceride
MCT: medium-chain triglyceride
MCT8: medium-chain triglyceride with 8 carbons
MCT10: medium-chain triglyceride with 10 carbons
NB: neuroblastoma
OXPHOS: oxidative phosphorylation
