# Supplementary material for: A ketogenic diet supplemented with medium-chain triglycerides enhances the anti-tumor and anti-angiogenic efficacy of chemotherapy on neuroblastoma xenografts in a CD1-nu mouse model
Source: Oncotarget. 2017 Aug 8;8(39):64728–44. doi: 10.18632/oncotarget.20041 (PMC5630289; doi:10.18632/oncotarget.20041)
Supplement: Supplementary file 1 [file oncotarget-08-64728-s001.pdf]

A ketogenic diet supplemented with medium-chain triglycerides enhances the anti-tumor and anti-angiogenic efficacy of chemotherapy on neuroblastoma xenografts in a CD1-nu mouse model

SUPPLEMENTARY MATERIALS

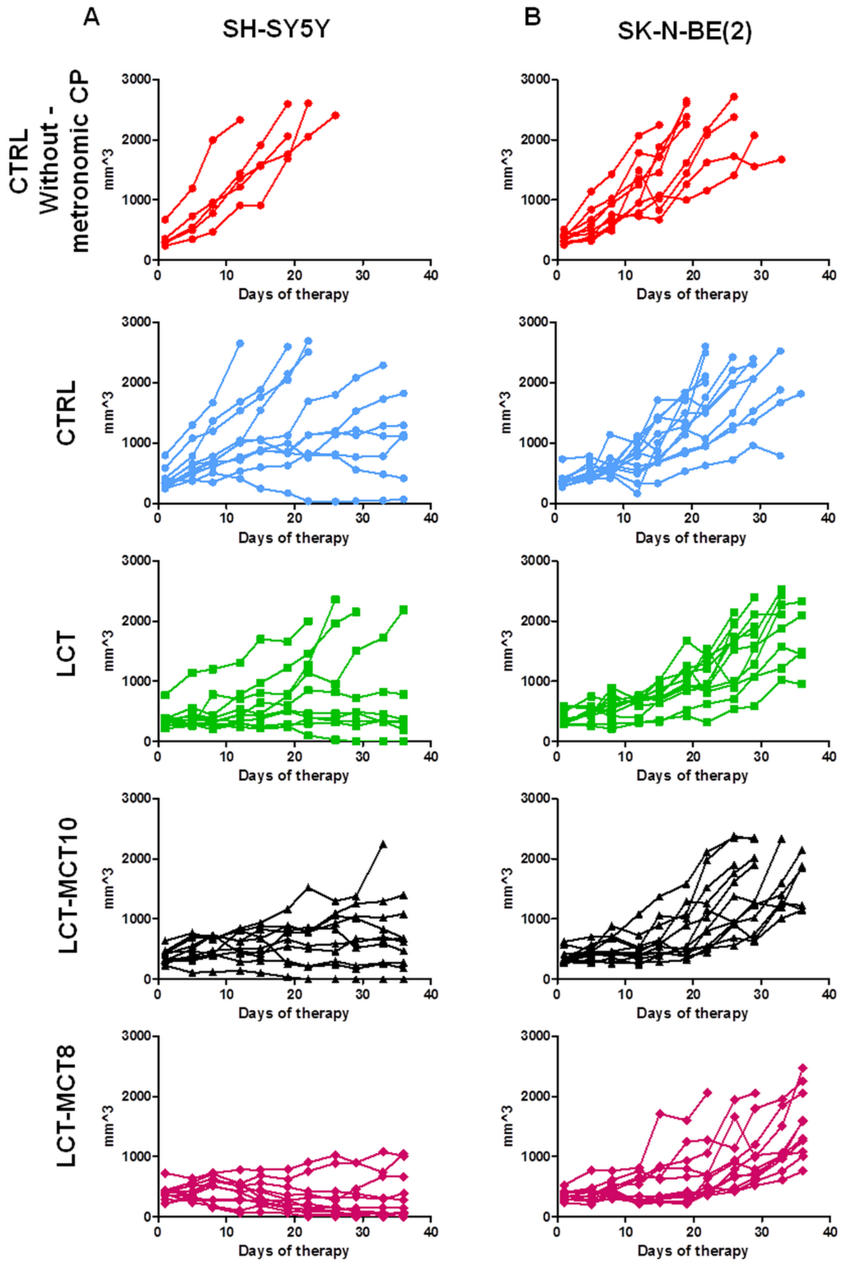

Supplementary Figure 1: Growth rates of (**A**) SH-SY5Y and (**B**) SKNBE(2) individual xenografts**.** The graphs show single tracks of tumor growth in different therapy groups.

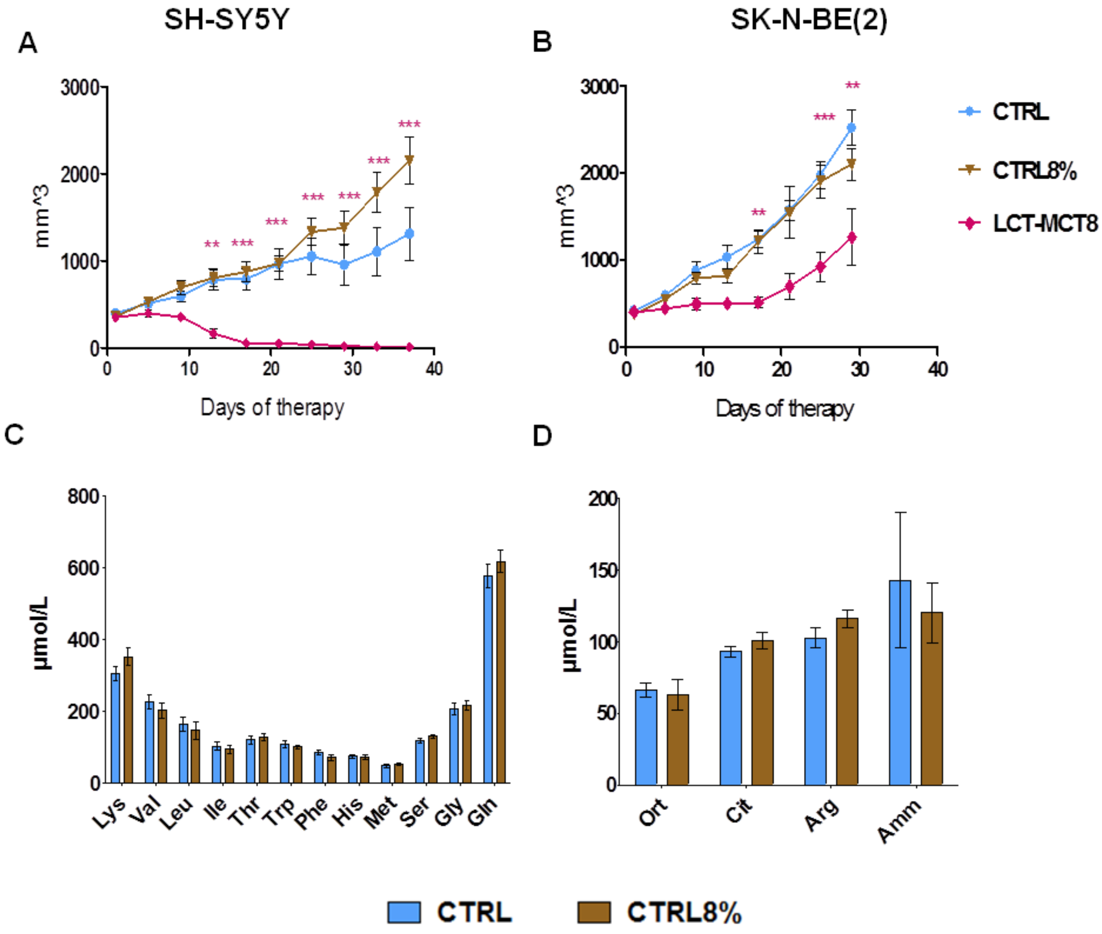

Supplementary Figure 2: NB growth and plasma amino acid levels were not affected by lower protein content in the diet. (**A**) SH-SY5Y and (**B**) SK-N-BE(2) growth curves show no growth inhibition by CTRL-8% compared to the CTRL diet, whereas significant NB growth suppression by LCT-MCT8 (n = 5) versus CTRL-8% (n = 10) and CRTL (n = 9) is observed. (C, D) Measurements of amino acids and urea and/or nitric oxide cycle metabolite levels in plasma of mice with SH-SY5Y xenograft revealed no influence of the protein content in the CTRL-8% versus the CTRL diet. Values are given as mean ± SEM (amino acid measurement: n= 8). One-way ANOVA followed by Dunnett's Multiple Comparison Test; ** $p \leq 0.01$; *** $p \leq 0.001$; LCT-MCT8 in combination with metronomic CP vs CTRL in combination with metronomic CP.

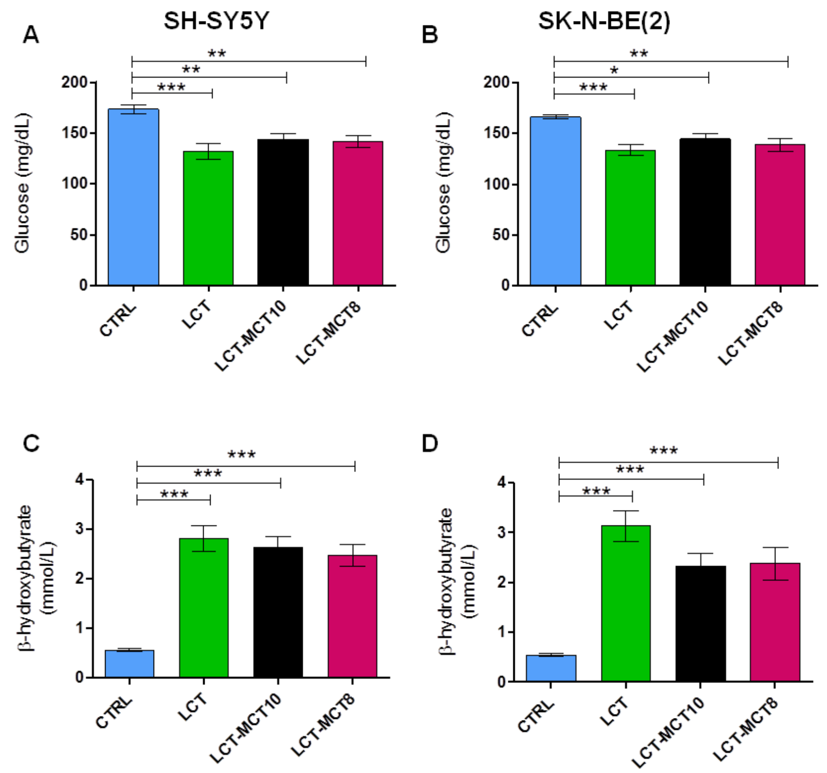

Supplementary Figure 3: The mean blood glucose **(A, B)** and BHB levels **(C, D)** in mice with **(A, C)** SH-SY5Y and **(B, D)** SK-N-BE(2) xenografts**.** Values are given as mean ± SEM (CTRL, LCT and LCT-MCT10 n = 10-12; LCT-MCT8 n = 11-12). One-way ANOVA followed by Dunnett's Multiple Comparison Test; *$p \leq 0.05$; **$p \leq 0.01$; ***$p \leq 0.00$; KDs in combination with metronomic CP vs CTRL in combination with metronomic CP.

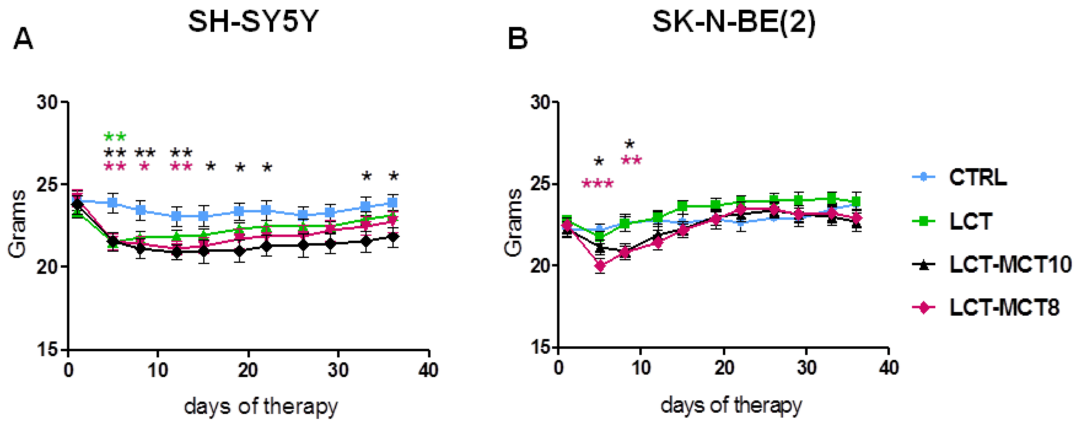

Supplementary Figure 4: Average body weight for mice with **(A)** SH-SY5Y and **(B)** SK-N-BE(2) xenografts. Values are given as mean ± SEM (CTRL, LCT and LCT-MCT10 n = 10-12; LCT-MCT8 n = 11-12). One-way ANOVA followed by Dunnett's Multiple Comparison Test; *p ≤ 0.05; ***p* ≤ 0.01; ****p* ≤ 0.001; KDs in combination with metronomic CP vs CTRL in combination with metronomic CP. Mouse body weight was calculated by subtracting the tumor weight from the whole-body weight.

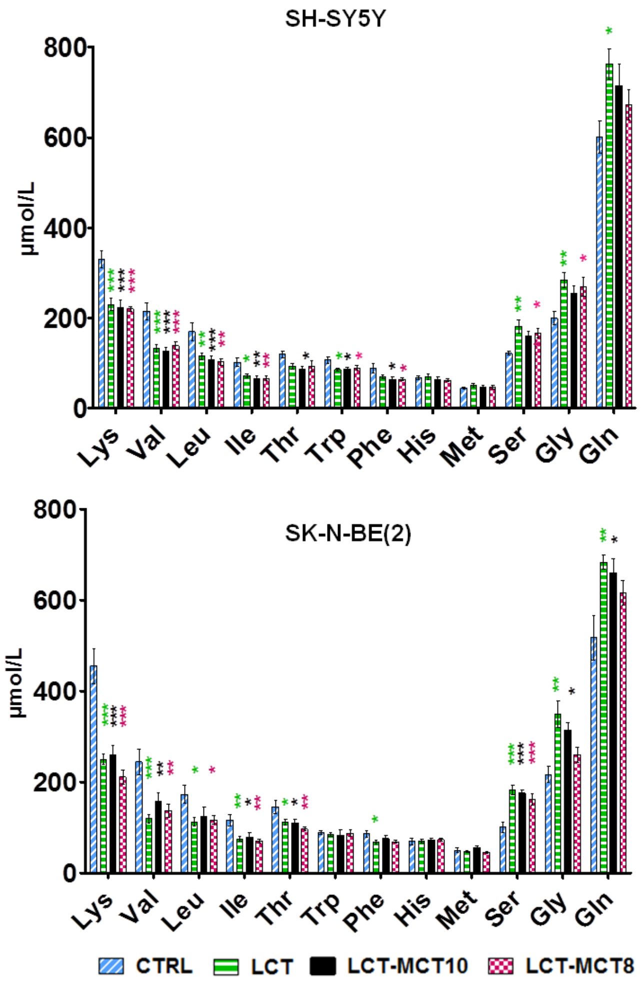

Supplementary Figure 5: Amino acids levels in plasma of mice with **(A)** SH-SY5Y and **(B)** SK-N-BE(2) xenografts. Values are given as mean ± SEM (n = 8). One-way ANOVA followed by Dunnett's Multiple Comparison Test; **p* ≤ 0.05; ***p* ≤ 0.01; ****p* ≤ 0.001; KDs in combination with metronomic CP vs CTRL in combination with metronomic CP.

Supplementary Table 1: Composition and calorie count of the diets. Mouse body weight (g) (mean ± SEM) for mice bearing SH-SY5Y and SK-N-BE(2) xenografts in the different therapy groups.

	CRLT	CTRL-8%	LCT	LCT-MCT10	LCT-MCT8
**Diets composition**					
	%	%	%	%	%
Crude Protein	16.1	8.1	8.1	8.1	8.1
LCT[a]	7.1	7.1	74.6	49.6	49.6
C8[b]	0	0	0	0	25
C10[b]	0	0	0	25	0
Sugar	6	6	1	1	1
Starch	51.2	60	0	0	0
Crude fiber	10	10	9.9	9.9	9.9
Crude ash	4.5	4.5	4.4	4.4	4.4
	per kg	per kg	per kg	per kg	per kg
Energy MJ	15.1	15.1	29.7	29.7	29.7
Vitamin A (IU/IE)	15	15	15	15	15
Vitamin D3 (IU/IE)	1.5	1.5	1.5	1.5	1.5
Vitamin E (mg)	150	150	150	150	150
Vitamin K3 (mg)	20	20	20	20	20
Vitamin C (mg)	30	30	30	30	30
**Mouse body weight (n = 22 - 23)**					
Initiation day	23.5 ± 0.4	23.5 ± 0.3	23.4 ± 0.2	23.4 ± 0.4	23.6 ± 0.3
Termination day (– tumor weight)	23.1 ± 0.3	23.0 ± 0.3	23.3 ± 0.3	22.1 ± 0.3	22.8 ± 0.4

CTRL, control; CTRL-8%, control with 8% protein; LCT, long chain triglyceride; MCT8, 8-carbon medium-chain triglyceride; MCT10, 10-carbon medium-chain triglyceride

[a]LCT composition: butter fat 11.7% and pork lard 88.3%

[b]MCT10 and MCT8 composition: pure oil

Supplementary Table 2: Blood parameters: blood glucose (mg/dl) / ketone (mmol/l) level, glucose-ketone index averaged from day 5 after the start of dietary intervention to the termination day, and amino acid levels (µmol/l) on the termination day for mice bearing SH-SY5Y and SK-N-BE(2) xenografts in the different therapy groups.

	CTRL	LCT	LCT-MCT10	LCT-MCT8
	Mean ± SEM	Mean ± SEM	Mean ± SEM	Mean ± SEM
**Blood glucose/ketone (n = 22 - 23)**				
Glucose	170.3 ± 1.9	132 ± 6.7	143 ± 5.6	140 ± 6.7
Ketone	0.5 ± 0.02	3.2 ± 0.1	2.6 ± 0.2	2.6 ± 0.1
Glucose-ketone index	20.8 ± 0.9	2.5 ± 0.2	3.6 ± 0.2	3.4 ± 0.2
**Plasma amino acids (n = 16)**				
Aspartic acid	10.5 ± 2.5	14.3 ± 1.5	13.8 ± 1.2	11.2 ± 1.0
Threonine	133.0 ± 8.8	101.4 ± 5.1	98.3 ± 5.6	95.4 ± 6.4
Serine	111.8 ± 6.6	182.2 ± 8.9	168.4 ± 6.1	164.5 ± 8.0
Asparagine	38.9 ± 3.2	43.6 ± 2.4	45.4 ± 3.6	41.3 ± 2.9
Glutamic acid	30.7 ± 6.6	36.6 ± 5.6	31.6 ± 6.0	26.8 ± 3.9
Glutamine	559.4 ± 31.3	725.6 ± 21.1	687.9 ± 27.7	647.6 ± 23.0
Proline	104.3 ± 13.0	117.2 ± 9.2	120.4 ± 13.3	83.1 ± 7.8
Glycine	208.4 ± 11.4	312.5 ± 17.6	285.4 ± 13.1	265.1 ± 13.3
Alanine	504.7 ± 47.0	581.3 ± 43.7	630.1 ± 65.8	509.4 ± 41.9
Citrulline	79.7 ± 5.6	117.2 ± 5.7	123.4 ± 7.2	114.6 ± 5.8
Valine	229.7 ± 16.9	126.2 ± 6.0	142.2 ± 10.3	138.0 ± 7.9
Cysteine	30.2 ± 4.5	27.8 ± 5.1	41.0 ± 7.4	32.3 ± 5.8
Methionine	47.6 ± 3.2	49.4 ± 2.7	50.9 ± 3.2	46.6 ± 2.5
Isoleucine	109.3 ± 7.8	73.9 ± 3.3	72.2 ± 6.4	68.6 ± 3.9
Leucine	171.6 ± 13.5	114.4 ± 5.5	116.2 ± 11.2	109.3 ± 6.3
Tyrosine	86.1 ± 8.4	66.4 ± 4.0	63.8 ± 4.4	60.8 ± 3.2
Phenylalanine	87.5 ± 5.7	69.0 ± 2.6	70.2 ± 4.0	66.3 ± 2.4
Ornithine	69.4 ± 4.6	50.3 ± 1.8	49.8 ± 3.2	47.0 ± 2.2
Lysine	401.8 ± 28.6	240.3 ± 8.8	246.2 ± 14.0	215.3 ± 9.0
Histidine	68.8 ± 4.6	70.2 ± 3.3	68.2 ± 3.7	67.3 ± 2.7
Arginine	104.8 ± 4.2	112.4 ± 4.8	108.5 ± 6.1	101.6 ± 4.7
Ammonia	177.3 ± 19.9	164.1 ± 16.2	147.0 ± 20.1	131.2 ± 12.6
Tryptophan	97.8 ± 4.5	85.5 ± 2.4	85.7 ± 5.4	89.0 ± 4.2

CTRL, control; LCT, long-chain triglyceride; MCT8, 8-carbon medium-chain triglyceride; MCT10, 10-carbon medium-chain triglyceride

Supplementary Table 3: Amino acid levels (µmol/kg) for SK-N-BE(2) tumors in the different therapy groups.

	CTRL	LCT	LCT-MCT10	LCT-MCT8
**Tumor amino acids (n=8)**				
Aspartic acid	2363.5 ± 168.8	2026.7 ± 270.8	2555.7 ± 188.7	2448.2 ± 254.4
Threonine	1096.5 ± 79.4	582.5 ± 59.9	738.5 ± 55.8	758.9 ± 80.6
Serine	656.7 ± 28.1	726.9 ± 100.4	797.7 ± 51.5	693.9 ± 46.9
Asparagine	343.6 ± 22.4	284.2 ± 28.3	370.8 ± 53.3	312.1 ± 38.3
Glutamic acid	3733.6 ± 261.6	3013.3 ± 407.2	3933.9 ± 381.2	3635.6 ± 402.8
Glutamine	1402.7 ± 147.4	1513.5 ± 225.4	2062.3 ± 205.1	1774.8 ± 218.0
Proline	1345.5 ± 125.6	1704.4 ± 230.6	1797.8 ± 221.4	1925.1 ± 470.3
Glycine	4228.9 ± 364.2	4448.4 ± 566.3	5032.1 ± 377.9	4495.0 ± 294.0
Alanine	4857.5 ± 351.4	4588.8 ± 571.2	5968.1 ± 463.2	5458.4 ± 419.6
Citrulline	166.7 ± 33.5	310.7 ± 35.2	364.5 ± 38.4	371.3 ± 32.5
Valine	534.0 ± 42.0	244.5 ± 23.5	374.5 ± 38.8	374.7 ± 38.4
Cysteine	25.7 ± 4.1	22.3 ± 3.2	28.7 ± 6.6	53.8 ± 18.3
Methionine	136.5 ± 10.0	104.7 ± 15.3	151.3 ± 19.7	93.2 ± 23.3
Isoleucine	240.4 ± 20.5	125.8 ± 16.4	165.5 ± 15.8	162.3 ± 21.0
Leucine	483.2 ± 36.7	264.0 ± 22.9	322.8 ± 22.7	312.3 ± 41.2
Tyrosine	274.5 ± 22.1	229.9 ± 19.9	244.8 ± 21.4	267.8 ± 41.6
Phenylalanine	185.4 ± 37.4	173.5 ± 16.9	204.4 ± 16.0	230.2 ± 35.8
Ornithine	112.4 ± 16.8	77.1 ± 12.2	69.9 ± 10.1	68.8 ± 9.4
Lysine	641.8 ± 25.3	434.5 ± 38.1	447.1 ± 33.5	417.9 ± 48.7
Histidine	238.4 ± 19.7	195.2 ± 27.7	241.2 ± 18.3	238.0 ± 30.8
Arginine	171.5 ± 16.9	136.6 ± 26.3	185.7 ± 20.2	176.3 ± 17.5
Ammonia	1189.2 ± 153.3	888.1 ± 83.5	809.0 ± 72.6	758.3 ± 97.6
Argininosuccinate	0.0	24.9 ± 9.7	46.9 ± 12.4	43.4 ± 10.2

CTRL, control; LCT, long-chain triglyceride; MCT8, 8-carbon medium-chain triglyceride; MCT10, 10-carbon medium-chain triglyceride
